# Mapping Digital Nudges and Recommender Systems for Obesity Prevention: Scoping Review

**DOI:** 10.2196/73151

**Published:** 2026-04-17

**Authors:** Sarah Forberger, Lucia A Reisch, Pieter Van Gorp, Christoph Stahl, Lara Christianson, Jihan Halimi, Karina Karolina De Santis, Chungwan Lo, Cassandra A Omane, Alejandra Loyola-Leyva, Laurent Malisoux, Tiziana de-Magistris, Torsten Bohn

**Affiliations:** 1 Leibniz Science Campus Digital Public Health (LSC DiPH), Bremen, Germany; 2 Leibniz Institute for Prevention Research and Epidemiology - BIPS, Achterstraße 30, Bremen, 28359, Germany, 49 42121856907, 49 42121856941; 3 El-Erian Institute for Behavioural Economics and Policy, Cambridge Judge Business School, Cambridge, United Kingdom; 4 Eindhoven University of Technology, Eindhoven, The Netherlands; 5 Luxembourg Institute of Science and Technology, Belvaux, Luxembourg; 6 Agro-Food Research and Tecnological Center of Aragon (CITA), Zaragoza, Spain; 7 Luxembourg Institute of Health, Strassen, Luxembourg

**Keywords:** recommender systems, lifestyle, physical activity, sedentary behavior, obesity prevention, digital health, digital intervention, digital nudges

## Abstract

**Background:**

Recommender systems are pivotal in organizing information to enhance noticeability, reduce overload, and streamline decision-making. They can be even more effective if combined with digital nudges. Digital nudging is a subtle approach that combines design, information, and interaction elements to create a choice architecture that can guide user behavior in digital environments. While promising in many fields, there is a notable gap in health promotion, particularly because digital nudges and recommender systems can encourage and support sustained healthier choices in nutrition, physical activity (PA), and sedentary behavior reduction to prevent overweight and obesity.

**Objective:**

This scoping review addresses these gaps by exploring how digital nudges and recommender systems are used in obesity prevention.

**Methods:**

We prospectively published the scoping review protocol and adhered to the PRISMA-ScR (Preferred Reporting Items for Systematic Reviews and Meta-Analyses extension for Scoping Reviews) guidelines. Eligibility was defined using the PCC (Population, Concept, Context) framework. We searched 7 bibliographic databases (MEDLINE and PsycINFO via Ovid, Web of Science, CINAHL via EBSCO, Scopus, ACM Digital Library, and IEEE Xplore) up to October 2023. Following a 2-stage screening by independent reviewers, we selected 68 articles that included 94 user evaluations.

**Results:**

Most articles (36/68, 53%) report on recommender systems focused on nutrition, with fewer (16/68, 23%) aiming to promote PA. Most studies on digital nudges (11/68, 16%) targeted nutrition-related nudges for shopping and meal selection (8/68, 2%). Articles address PA and sedentary behavior less frequently (3/68, 4%). Three out of 68 (4%) articles report on recommender systems in combination with games, and 2 out of 68 (3%) articles report on recommender systems and digital nudges. Approaches to item retrieval vary widely, with 31 out of 68 (46%) articles failing to describe their methods. In the scoping review, we found a discrepancy between the target group for which the system was developed and the group with which the evaluation was conducted. Sixty-eight evaluations report positive results, while 26 studies report mixed, negative, or no-difference results.

**Conclusions:**

Integrating digital nudges and recommender systems might hold potential in overweight and obesity prevention by subtly encouraging healthier lifestyle choices. However, the heterogeneity in study designs, outcome measures, and reporting quality limits the comparability of findings and makes it difficult to draw robust conclusions about effectiveness. Future work should include detailed definitions, mechanism descriptions, broader geographic representation, and rigorous intervention testing and user evaluations to fully leverage these systems for improved health outcomes and to support sustainability and well-being objectives.

## Introduction

### Background

Recommender systems are tools designed to predict and suggest items, services, or content that users might like based on their preferences, behavior, or other data. For instance, recommender systems can suggest recipes informed by the user’s past experiences and dietary preferences, such as vegan or vegetarian options. These preferences are discerned by analyzing and comparing the search histories of different users [1,2]. Furthermore, these systems can remind users to increase their physical activity (PA), integrate tasks into daily schedules based on personal calendars and to-do lists, calculate caloric expenditure, and propose recipes based on that expenditure, while also suggesting suitable exercise methods [3]. In this process, recommender systems use sophisticated algorithms to analyze extensive datasets. This approach facilitates tailored suggestions by applying diverse filtering techniques [4-6]. Therefore, recommender systems are considered digital interventions that leverage nudging mechanisms in the digital environment [7]. Digital nudging is an approach grounded in behavioral economics that utilizes user interface design elements to influence user choices in digital environments. These elements encompass, for example, graphic design, coloring, specific content, wording, ranking, and other features, collectively serving as subtle tools to guide individuals toward decisions or behaviors [8]. In other words, they provide a choice architecture in the digital space intended to guide individual decisions. The digital environment is where users make decisions on screens, such as tablets, websites, or mobile apps [9]. Nudging mechanisms, according to the taxonomy developed by Münscher et al [10] and reflected in the functionality of recommender systems, can be categorized into:

- Decision information: This refers to techniques that target the presentation of decision-relevant information without altering the decision options. This category focuses on how information is perceived, represented, and framed, influencing the decision-making process. It includes methods such as translating information to make it clearer, reframing choices to alter perspectives, simplifying complex data to enhance comprehension, and making critical information or social norms visible to guide decision-makers without changing the underlying content.
- Decision structure: This involves altering the arrangement and presentation of decision options to influence decision-making strategies and outcomes. This approach includes modifying the decision environment by setting defaults, adjusting the range or composition of available options, changing the effort required to select an option, and altering the potential consequences associated with each choice. The goal of these techniques is to guide decision-makers toward more desirable outcomes by organizing and structuring the decision space in a way that impacts their selection process.
- Decision assistance: This encompasses techniques aimed at supporting individuals in following through with their intentions to bridge the intention-behavior gap. This category includes interventions such as reminders to bring desired options to the forefront of attention and facilitating commitment to reinforce adherence to chosen behaviors. These techniques are designed to enhance self-regulation and assist decision-makers in maintaining their resolve, thereby increasing the likelihood of consistent and intended actions.

Recommender systems automatically select information that influences information noticeability [10]. They sort information based on preferences and prompt as suggestions to elicit a reaction from users. In doing so, recommender systems can reduce information overload, help rank information, and streamline the decision-making process [1,2].

While recommender systems inherently possess features such as information selection, simplification, and reminders akin to digital nudges, they are rarely included in the category of digital nudges in the field of health promotion. They are designed with these principles in mind. Interactions within these systems, such as visual feedback on daily step counts, calorie tracking, messages, and reminders, are infrequently developed with the explicit framework of digital nudges as the guiding principle. By effectively using design, information, and interaction elements, digital nudges aim to steer decision-making processes. Combining digital nudges and recommender systems could increase their individual effectiveness, as demonstrated in various studies [8,11-13]. However, only a small number of nudges with recommender system mechanisms have been investigated until now [7], and large gaps exist [14]. Furthermore, the literature is characterized by expanding research activity, volume, and heterogeneity [15].

However, given the effects that digital nudges and recommender systems can exert, this potential should be leveraged for obesity prevention, a crucial aspect of modern lifestyle interventions. According to Hummel et al [16], 62% of nudging treatments have statistically significant effects, with a median effect size of 21%, which varies by category and context. Defaults emerge as the most effective strategy (number of included studies: 21, median effect size: 50%; average effect size: 87%), while precommitment strategies are the least effective (number of included studies: 2, median effect size: 7%; average effect size: 7%). Additionally, digital nudging demonstrates comparable effectiveness and presents opportunities for individualization, allowing for more tailored approaches to influencing healthy behaviors. Obesity prevention has become an important part of public health prevention due to the drastic increase in obesity in the past decades, with adult obesity rates as high as 34% in some European countries and even 40% in the United States [17]. As the trends continue almost unencumbered, novel approaches are required to prevent and reduce obesity, and digital tools have aimed to contribute to this. Utilizing recommender technologies and nudging strategies aids users in navigating the extensive and often heterogeneous data landscape. This approach is instrumental in identifying accurate and relevant information essential for making informed health-related decisions [18-20]. By using a personalized digital choice architecture, the way information, services, and choices are presented to users on websites and other online services can be altered. This approach can subtly encourage desired behaviors such as improved dietary habits to prevent weight gain and enhance metabolic health. Additionally, it can promote more physically active lifestyles and support maintaining healthy weights. Research into digital nudging analyzed how users perceive choices and make decisions in digital environments [7,8]. Combining this knowledge with recommender systems allows for presenting tailored suggestions within a choice architecture that naturally guides users toward desirable decisions. For example, combining healthy food items based on user preferences with visual progress bars or social comparison features (eg, “90% of users chose this option”) could encourage users to follow recommendations [21,22]. General challenges reported by individuals who are gaining weight, such as making poor choices when tired or hungry, focusing on budget constraints rather than a balanced diet, lacking time to gather information on healthy food items or exercise, or staying motivated over a more extended period, can be addressed by both approaches.

Bergman et al [13] described various types of digital nudges based on digital nudge patterns, outcomes, context, evaluation, personalization, interconnectivity, and mode of delivery. However, the context was broadly labeled as “health,” without specifically addressing the behavior targeted by these nudges. Similarly, Jesse and Jannach [7] developed a taxonomy for coding digital nudges, integrating the work of others by categorizing nudges and their mechanisms [10,22-24]. However, their work did not focus on the health context of the systems. A similar pattern can be seen in the literature on recommender systems. De Santis et al [25] identified 10 reviews from 2017 to 2023, covering 308 primary studies on recommender systems and nudges for obesity prevention, primarily focusing on technical system properties and strengths and weaknesses [1,26-35]. While health domains related to obesity prevention were mentioned, system implementation and evaluation with users were rarely described. De Croon et al [28], for example, analyzed the use of recommender systems in health applications aimed at laypersons and identified 26 nutrition-related recommender systems, including meal plans, recipes, restaurant suggestions, and fruits. Further, 24 studies were included under lifestyle, encompassing PA and the combination of nutrition and PA, as well as other lifestyle behaviors such as smoking. Similarly, Pincay et al [6] classified various studies analyzing recommender systems under “wellness,” which covered diets, exercise routines, and care recommendations for children and older people. Pincay et al [6] did not distinctly separate nutrition from PA behaviors. Cai et al [2] identified 24 health domains. The health domain (specific health conditions, issues, or diseases) identified ranges from general healthy lifestyle promotion to specific health domains such as diabetes and obesity. Bondevik et al [1] analysis focused on technical aspects of food recommender systems, such as methods and techniques used within the recommender system (eg, filtering, personalization) data processing techniques (eg, data used for calculation similarities, data used for training the system), evaluation, and reproducibility without considering specific target groups, topics, or the integration of approaches such as digital nudges. Sun et al [36] offered a broader perspective, mapping health recommender systems based on characteristics, techniques, interfaces, and user involvement. However, health behaviors were broadly categorized under “lifestyle.” No review has explicitly focused on nutrition, PA, and sedentary behavior (SB) reduction. Furthermore, given the diversity of contexts in which health behaviors occur, it is crucial to consider socioeconomic and geographical disparities, as these can significantly impact mechanisms, norms, acceptance, effectiveness, and, in the end, the generalizability of digital intervention strategies. More information is needed on current research approaches, target audiences, and employed methods and techniques to efficiently use recommender systems, digital nudges, or a combination of both for overweight and obesity prevention.

### Study Aims and Objectives

This paper addresses the gaps in mapping how digital nudges and recommender systems are currently used in research to target overweight and obesity by addressing nutrition, PA, and SB reduction.

The detailed objectives of this scoping review are as follows:

1. To identify digital nudges or recommender systems for overweight and obesity prevention that target diet, PA promotion, or SB reduction.
2. To map the digital nudges and recommender systems according to the target population, health behavior (diet, PA, or SB), and system classification (eg, mechanisms for developing recommendations, delivery channels, personalization, interconnection, and used combinations).

In doing so, our goal is to identify the current state of research, identify gaps, and identify the next steps in research. By understanding these gaps, researchers can better identify what needs to be addressed in the next steps and help avoid repeating approaches.

## Methods

### Study Design, Protocol, and Registration

A detailed description of the methods for this scoping review was previously published [37], and this scoping review follows the PRISMA-ScR (Preferred Reporting Items for Systematic Reviews and Meta-Analyses extension for Scoping Reviews) guidelines [38]. We chose a scoping review because it allows us to map the key concepts and gaps within the area of interest. The work on this study began in July 2023, and the database searches were conducted in October 2023. Screening and study selection were conducted from October 2023 to March 2024, data extraction from April to October 2024, and data synthesis from November 2024 to February 2025.

### Ethical Considerations

No ethical approval was required for this review, as the data were obtained from publicly available materials.

### Eligibility

The eligibility criteria were based on the PCC (Population, Concept, Context) framework [39]. We included primary studies of any design (randomized or nonrandomized, with quantitative or qualitative data) (Table 1). We excluded all articles that tested the system using hypothetical or predefined datasets, focusing only on studies that tested it on a real-world sample.

**Table 1.** Eligibility for inclusion in the scoping review.

PCC^a^	Inclusion criteria	Exclusion criteria
Population	Any human population groups (children or adults; healthy, at risk for chronic diseases, or clinical samples)	Studies that do not test the systems or nudges with humans, and studies that only use hypothetical or predefined datasets.
Concept	Digital nudges or recommender systems (stated in the title, abstract, or full text of a study)	Digital nudges or recommender systems not used, or studies that use digital nudges or rely on recommender systems but do not name them in the title, abstract, or full text
Context	Any geographical setting; overweight and obesity prevention (eg, nutrition, food recipes, grocery stores, meal preparation, PA^b^ promotion, SB^c^ prevention)	Digital nudges or recommender systems used in fields, such as blockchain, finance, security, privacy, agriculture, service, and e-commerce rather than for overweight and obesity prevention

a PCC: Population, Concept, Context.

b PA: physical activity.

c SB: sedentary behavior.

As PA and SB are not opposites but complementary, we included studies targeting PA and SB. PA is defined as any bodily movement generated by skeletal muscle contraction that increases energy expenditure above the resting metabolic rate and is characterized by its modality, frequency, intensity, duration, and context of practice [40]. In contrast, SB includes any waking behaviors characterized by an energy expenditure of ≤1.5 metabolic equivalents while in a sitting, reclining, or lying posture [41]. In general, this means that one can be both physically active (eg, meeting the WHO recommendations for PA) and highly sedentary (eg, accumulating a high amount of sitting time), which significantly influences the choice of intervention elements to be utilized, depending on the targeted specific behavior [42].

### Information Sources and Search Strategy

The search strategy was developed with the assistance of an information specialist, in collaboration with the involved experts (coauthors). We utilized the Joanna Briggs Institute (JBI) 3-step approach [43] to formulate the search strategy using keywords as well as index and MeSH terms [44]. The seven databases searched until October 2023 were MEDLINE and PsycINFO via Ovid, Web of Science, CINAHL via EBSCO, Scopus, ACM Digital Library, and IEEE Xplore. The full search strategy for Medline can be found in Multimedia Appendix 1.

### Study Selection

Two reviewers independently screened the records using predefined eligibility criteria based on the PCC framework (Table 1) in a 2-stage process. Titles and abstracts were screened in Rayyan, followed by a full-text screening in Covidence. Any disagreements were resolved through discussion at each stage between the 2 reviewers or through the involvement of a third reviewer.

### Data Charting and Data Items

A team of reviewers performed data extraction using a standardized, self-developed, and pilot-tested extraction form. After extraction, one researcher (SF) processed the data into predefined categories and verified the coding with a second team member (Table 2).

**Table 2.** Predefined categories.

Code	Operationalization	Explanation
Country/region	Country: as indicated in the publication, if not indicated in the publication, the country of the first author’s affiliation	High-income, upper-middle-income, lower-middle-income, and lower-income countries based on the World Bank country classifications by income level (2022‐2023)
Recommender system classification [45]	Methods: approach that the recommender system uses to perform the retrieval of items [6]	Content-based filtering, where recommendations are made by identifying items similar to those previously liked by the user or other users with similar profiles [46,47]. Collaborative filtering, which relies on similarities between users to recommend items based on the preferences of other users with similar profiles [48]. Hybrid recommendation combines multiple techniques to overcome the limitations of single approaches [45]. An example is the deep learning approach to item retrieval in recommender systems that refer to the application of deep neural networks (deep learning) to filter relevant items (eg, products, films, songs) for a user from a vast pool of options. Deep learning is used in this context to uncover complex patterns within data, thereby enabling more precise and personalized recommendations. Knowledge-based recommendation is another approach based on user preferences and constraints [35]. Collecting the preferences of a user (preference elicitation) within the scope of a dialogue and then recommending items either (1) based on a predefined set of recommendation rules (constraints) or (2) using similarity metrics that help to identify items which are similar to the preferences of the user [35]. Evolutionary approaches rely on optimization algorithms inspired by natural evolution to retrieve or rank items. These approaches are beneficial when the search space for items is vast, and the system needs to explore a variety of combinations to find the most optimal recommendations.
	Techniques	How the relationships between items are computed (eg, Rasch-tailoring, TOPIs^a^, conversational agent, iALS^b^ algorithm) [6].
Age	Children (0‐14), 15‐17, 18‐40, 40‐65, adults (18-65), ≥60, older adults (≥65), adults (18-65) and older adults (≥65), not reported	Where possible, the mean age was used to report age data. If the mean age was not provided, age ranges were used, and the target group was assigned accordingly based on these age ranges.
Outcome	Positive: only positive results are reported Negative: only negative results are reported Mixed: positive and negative results are reported No difference	A qualitative assessment of the results was chosen to consolidate the extremely varied reporting of outcomes (such as significance levels, descriptive statistics, content analyses, and log data).

a TOPI: Topic Models Over Points of Interest.

b iALS: Implicit Alternating Least Squares.

### Critical Appraisal

No evidence appraisal was conducted. In a scoping review, the primary aim is to map the existing literature and provide an overview of the breadth and scope of research on a particular topic rather than to assess the quality of the evidence. This type of review typically includes a wide range of studies, often without applying strict inclusion criteria related to methodological rigor. As a result, scoping reviews do not appraise evidence quality, focusing instead on identifying and categorizing available evidence.

### Overlap of Primary Studies Included in This Review and Other Reviews With Systematic Methodology

In addition to this scoping review of primary studies, we conducted a second scoping review of reviews identified in our search [49]. The scoping review of reviews included 10 reviews (n=8 systematic reviews and n=2 scoping reviews). The reviews addressed recommender systems for any human populations (n=6), described the systems (n=8), and focused on nutrition (n=6). The overlap between the primary studies included in the 10 reviews and those in this review was low. Specifically, of the 308 primary studies included in the 10 reviews, 10 primary studies were included in this scoping review.

## Results

### Characteristics of Included Studies

**Figure 1.**
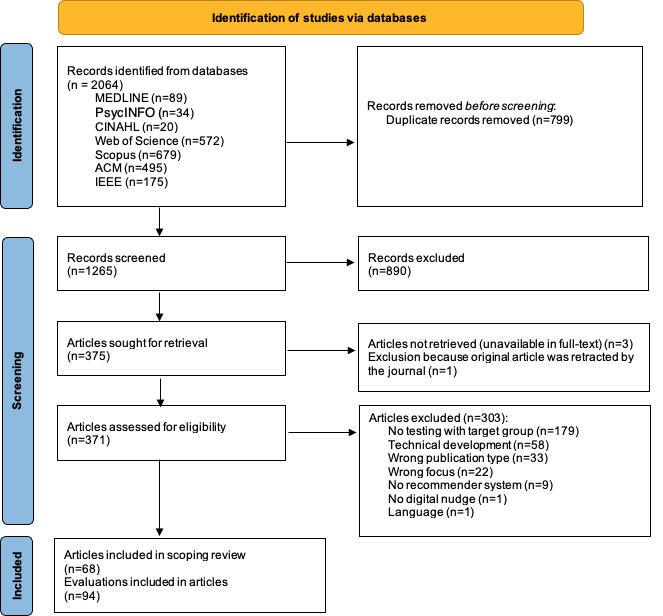
PRISMA (Preferred Reporting Items for Systematic Reviews and Meta-Analyses) flow diagram.

**Figure 2.**
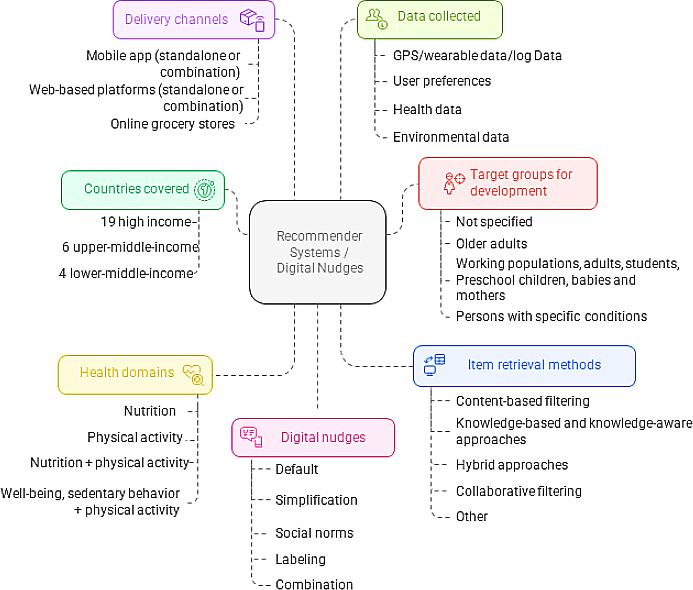
Overview of the results: study characteristics, behaviors addressed, item retrieval methods, and digital nudges used.

Of the 2064 records identified, 375 full-text articles were reviewed, and 68 articles covering 94 user evaluations were included (Figure 1). A detailed overview of the included studies is provided in Multimedia Appendix 2. Figure 2 provides an overview of the results.

### Countries or Regions

Based on the World Bank’s country classifications by income level (2022‐2023), the articles were published from 19 high-income countries (65%), 6 upper-middle-income countries (21%), and 4 lower-middle-income countries (14%). The United States was the most represented among the high-income countries [50-58], followed by Germany and the Netherlands [59-73], Belgium [74-79], Italy [80-84], and Portugal [85-88].

The study overview table in Multimedia Appendix 2 provides a detailed overview of the countries and regions covered, as well as the study characteristics.

### Target Population Addressed for Whom the Digital Nudges or Recommender Systems Were Intended

Among the 68 articles, the majority did not specify the target group for which the digital nudges or recommender systems were intended (52/68, 76%). For those mentioning a target population, older adults (≥65 y) were the most frequently mentioned group (3/68, 4%, Table 3) [86-88]. All other target groups, such as mothers with babies [89], students [71], and persons with specific conditions, were each addressed once (Table 3). The addressed conditions in the articles included diabetes (2/68, 3%) [90,91], congenital [70] and coronary heart disease [57], and hypertensive patients [92] (except for diabetes, each mentioned once).

**Table 3.** Target groups for which the recommender systems or nudges have been developed.

Target groups mentioned	N=68	References
Not specified	52	—^a^
Target groups mentioned		
Babies and mothers	1	[89]
Preschool children (3‐6 y)	1	[85]
Students	1	[71]
Adults	1	[93]
Working population	1	[94]
Older adults (≥65 y)	3	[86-88]
Persons with specific conditions		
Children with obesity	1	[95]
Adults with overweight and obesity	1	[53]
Diabetic patients	2	[91]
Hypertensive patients	1	[92]
Coronary heart disease patients	1	[57]
Patients with congenital heart disease	1	[70]
Insufficiently active people	1	[75]

a Not applicable.

### Behaviors Addressed by the Digital Nudges and Recommender Systems

The articles predominantly focused on dietary behaviors, with 54 out of 68 (79%) articles covering nutrition. PA was addressed in 22 out of 68 (32%) articles. One article explicitly included SB, in combination with PA and mental health [94]. Fifteen out of 68 (22%) articles reported on PA.

Eight out of 68 (12%) articles explored nutrition and PA together [53,56,85,93,96-99] (Table 4).

**Table 4.** Behaviors addressed within the articles.

Behavior	Articles, n (%)	References
Nutrition	54 (78)	—^a^
PA^b^	22 (32)	—
Behavior-specific nutrition		
Nutrition (meal choice)	30 (44)	RS^c^: [52,58,61-64,69,78,81-84,88,90,91,100-109]; mixed: RS + nudge: [63,110]; nudges: [65,69,108]
Nutrition (macronutrient composition)	13 (19)	RS: [45,50,66,67,72,80,87,89,92,111,112]; nudges: [73]
Nutrition (shopping)	7 (10)	RS: [51,79,113]; nudges: [55,59,77,114]
PA		
PA	15 (22)	RS: [54,57,60,68,74-76,86,115-117]; nudges: [70,71]; mixed (RS + game): [60]
Nutrition and PA		
Nutrition (macronutrient composition), PA	3 (4)	RS: [53,98]; mixed: RS + game: [93]
Nutrition (meal choice), PA	3 (4)	RS: [56,96,99]
Nutrition (eating, drinking), PA, sleeping	1 (1)	RS: [85]
Healthy living (diet, PA included)	1 (1)	Mixed: RS + game: [95]
PA and mental well-being		
PA, SB^d^, physical and mental well-being	1 (1)	Nudges: [94]

a Not applicable.

b PA: physical activity.

c RS: recommender system; mixed: articles using RS and nudges or RS and games.

d SB: sedentary behavior.

### System Characteristics (Recommender Systems, Nudges, Personalization, Interconnection, Delivery Channels)

**Figure 3.**
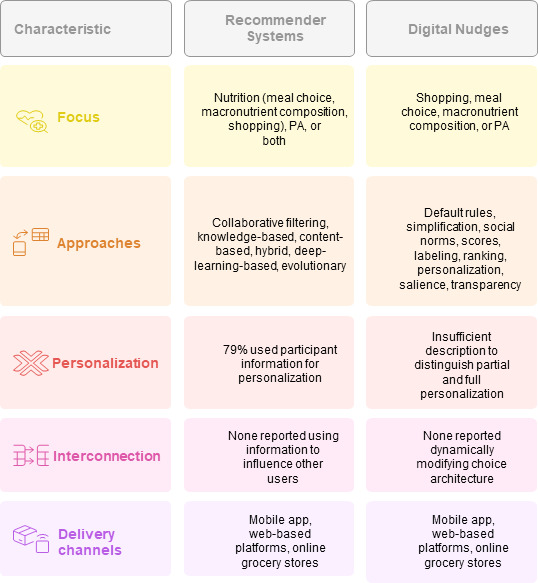
Characteristics: comparison between recommender systems and digital nudges. PA: physical activity.

Fifty-two out of 68 (76%) articles reported on recommender systems, 11 out of 68 (16%) articles focused on nudges [55,59,65,69-71,73,77,94,108,114] and 2 out of 68 (3%) articles discussed a combined system incorporating both a recommender system and a nudge [63,110]. Additionally, 3 out of 68 (4%) articles explored recommender systems in combination with games [60,93,95]. An overview of the characteristics can be found in Figure 3.

### Recommender Systems

In the category of recommender systems (52/68, 76%), 36 out of 68 (53%) articles addressed recommender systems targeting nutrition (meal choice: 22/68, 32%; macronutrient composition: 11/68, 16%; shopping: 3/68, 4%). Additionally, 16 out of 68 (23%) articles reported recommender systems used to increase PA, 10 out of 68 (15%) articles purely targeting PA, and 5 out of 68 (7%) articles examined a combination of nutrition and PA, with 1 article also incorporating sleep behavior.

The approaches used by the recommender system to perform the retrieval of items [6] were collaborative filtering (2/68, 3%) [62,100], knowledge-based and knowledge-aware approaches (5/68, 7%) [64,67,84,89,90], content-based filtering (6/68, 9%) [64,66,79,87,92,105], hybrid approaches (6/68, 9%) [83,95,102,103,113,117], as well as, for example, deep-learning-based [107] and evolutionary [96] approaches (1/68 each) (definitions: Table 2) and various combinations of the systems. In 22 out of 68 (32%) articles, the methods used within the recommender systems were not further specified. In another 9 out of 68 (13%) articles, while the recommender system itself was not clearly defined, the accompanying techniques used were described, including rule-based reasoning, conversational agents, artificial intelligence (AI)–based personalization, or Rasch-based tailoring.

### Digital Nudges

Within the nudge category (11/68, 16%), 4 articles focused on shopping behavior [55,59,77,114], and 3 out of 68 (4%) articles addressed meal choice [65,69,108]. One article focused on addressing macronutrient composition [73]. Additionally, 3 out of 68 (4%) articles targeted PA [70,71,94], with 1 placing PA within a broader context, including SB and physical and mental health [94].

In articles reporting on digital nudges targeting nutrition behavior, various nudges were tested: default rules (eg, automatic assignment to a category, default setting of the food group, or recipe class), simplification (eg, simplified representation using images, pictograms, or the display of labels), and social norms (information on the behavior of what most people have done) [59]; the combined use of scores such as Nutri-Score and Eco-Score (graphics) and social norms [77]; social norms alone [55]; ranking and personalization [65]; or a combination of different approaches (defaults, social norms, warnings, defaults, and social cues) [114]. Combinations of salience (stimuli that capture the attention and influence decision-making, such as attractive or unattractive looking unhealthy food products), transparency (adding a message about what the nudge is intended to do), self-nudging (giving individuals the opportunity to adjust the nudge according to their preferences or decide whether they want it to be instantiated) [69], social norms, hedonic goal nudges and positional nudges [108], and attentional bias [73] were also explored.

In the context of PA studies, the digital nudges tested included providing information [70] and motivational prompts [71].

### Personalization and Interconnection

Forty-one out of 52 (79%) recommender articles reported using participant information for system personalization including partial personalization (eg, preferences, likes, GPS, health data, wearable data, survey data). Questionnaires for user preferences were commonly used, with some studies also incorporating wearable data for partial or full personalization. However, the descriptions were insufficient to clearly distinguish between partial personalization (where a study gathers user data—such as location, demographics, or user actions—to infer the potential influence of the nudge/recommender system on user behavior) and full personalization (where information is used to personalize the choice architecture of individual users dynamically).

None of the studies reported using this information to influence how information from other users affects the analyzed user behavior (partial interconnection). Similarly, none of the studies provided information on how the actions of one user, in turn, dynamically modify the choice architecture of other users (full interconnection).

### Delivery Channels

Nineteen out of 68 (28%) articles did not provide information on the mode of delivery or the devices used to access the systems. Of these, 14 out of 68 (21%) were recommender system studies and 5 out of 68 (7%) were digital nudge studies. The most commonly reported delivery method was through a mobile app (27/68, 40%), either as a stand-alone application or in combination with a desktop application, a wearable, or a game. Web-based platforms, either as a standalone approach or in combination with an app, were reported in 11 out of 68 (16%) articles [50,55,68,79,84,102-104,106,111,114,118]. Online grocery stores were used in 3 out of 68 (4%) studies [59,69,77]. One study did not fit into these categories because it was conducted in fast-food restaurants using online menu boards [108].

### Evaluations Reported

#### Population on Which the Digital Nudges/Recommender Systems Were Tested

In the 68 included papers, 94 evaluations were reported. Most systems were evaluated with individuals aged 18 to 40 years (28/94, 26%), followed by adults aged 18 to 65 years (17/94, 18%). Further evaluation groups included combinations of adults aged 18 to 65 years and ≥60, 18 to 65 and ≥65, 18 to 40 and ≥65, and 40 to 65 years (altogether 12/94, 13%). Other individuals included were those aged 15 to 17 years, alone and in combination with other age groups (2/94, 2%), and those aged 0 to 14 years (2/94, 2%). In 23 out of 94 (24%) evaluations, the age range of the evaluation group was not reported. Furthermore, 53 out of 94 (78%) evaluations reported the gender of the evaluation group, with 51 (54%) reporting mixed gender and 2 (2%) reporting exclusively on women [89,93].

The sample sizes used for the evaluation varied substantially (Table 5). In 5 evaluations, no sample size was provided.

**Table 5.** Overview of the participants in the evaluations.

Sample size	Evaluations^a^						
	RS^b^			Digital nudges			RS + nudges, RS + game
	Nutrition	PA^c^	Mixed	Nutrition	PA	Mixed	
0‐10	[87,89,92]	[54,57,68,86]	[56,95,97,98]	—^d^	—	—	—
11‐20	[45,51,52,67,78,79,81,87,106,107]	[116]	[56]	[65]	—	—	RS + game, mixed behavior: [93,95]
21‐30	[50,88-91,101,109,111]	[75,76,115]	—	—	—	—	—
31‐40	[66]	[74,117]	[85]	—	—	—	—
41‐50	[82,102,105]	—	—	—	—	[94]	—
51‐100	[58,72,78,100,113]	—	[53,96]	[55,114]	[70,71]	—	RS + game, mixed behavior: [95]
101‐200	[51,61,64,84,104]	—	[99]	[108,114]	—	—	—
201‐300	[58,63,109]	—	—	[59,73]	—	—	—
301-	[62,64,83,112]	[60]	—	[69,77]	—	—	RS + nudges, nutrition: [110]
No reporting	[52,72,103]	—	—	—	—	—	—

a Some articles contain several evaluations.

b RS: recommender system.

c PA: physical activity.

d Not available.

Twenty-five out of 94 (27%) evaluations reported intervention time. The shortest intervention time reported was 7 days [92,97], while the longest was 3 years [95].

Twenty-five out of 94 (27%) evaluations reported an evaluation design. These designs included randomized controlled trials (7/94, 7%) [53,69-71,75,77,94], 2×2 between-subject designs (4/94, 4%) [62-64,104], online experiments (4/94, 4%) [55,59,73,109], single-case experiments [56], 2×2 mixed research designs [64,72], and quasi-experimental designs [57] (2/94, 2% each). Further designs were, for example, pre-post surveys [50], field experiments [59], longitudinal between-subject designs [76], effect differences between groups and within subjects [66], and 2×2 within-subject experiments [65].

The instruments used for evaluation ranged from questionnaires [51,67,81,87-89,95] and online surveys [60,78,91,96,110,113], focus groups [78], and ratings [61,82,84,95] to combinations of these methods with task performance, observations, gameplay monitoring, or user data.

#### Evaluation Results

This scoping review includes 94 evaluations, of which 68 report positive results. There are 26 studies with mixed, negative, or no-difference results. In the domain of recommender systems related to nutrition, there were 8 studies with mixed results [45,67,79,84,87,89,92,109], 3 with negative outcomes [61] (2 evaluations in [64]), and 1 showing no difference [52], totaling 12 studies. For recommender systems targeting PA, 4 studies reported mixed outcomes [54,68,76,116]. In the case of recommender systems combined with nudges, 1 study reported mixed results [110]. Two studies reported mixed outcomes when combining recommender systems and games [60,95]. Studies involving nudges showed mixed results across 7 evaluations [55,59,70,77,94,108,118], with 1 showing no difference [71]. It is important to note that further comparison is not feasible due to the extreme heterogeneity of approaches, methods used, outcomes, and research questions, ranging from, for example, user and system evaluation, user satisfaction evaluation, system or chat performance, choice satisfaction, plausibility tests, or intervention validations across these studies (Multimedia Appendix 2).

## Discussion

### Principal Findings

Recommender systems help users avoid information overload and aid in decision-making by using filtering techniques to suggest relevant content. These systems typically present users with a curated list of items tailored to their preferences or needs, simplifying the process of finding and evaluating relevant information. By doing so, they streamline decision-making and make information searching more efficient [10]. These approaches can be classified as nudges, allowing recommender systems to be grouped under the category of digital nudges. This scoping review aimed to identify digital nudges or recommender systems for overweight and obesity prevention targeting diet, PA promotion, or SB reduction and to map the digital nudges and recommender systems according to the target population, health behavior (diet, PA, or SB), and system classification (eg, mechanisms for developing recommendations, delivery channels, personalization, interconnection, and used combinations).

Recommender systems identified in this work predominantly focused on dietary behavior, with less emphasis on promoting PA. Additionally, the studies found that reporting on digital nudges primarily focused on nutrition, influencing shopping behavior and meal choices. Other health behaviors, such as PA and SB, are rarely reported. Only one study reported a combination of recommender systems and nudges. With this overview, the scoping review aligns with other reviews that reported that, in health promotion, the combination of recommender systems and nudges is underused. Jesse and Jannach [7] identified 9 domains in 18 articles that combined digital nudges and recommender systems. The domains were route planning, e-commerce, internet services, and social media, with route planning and e-commerce being the most popular ones.

This scoping review underscores the imbalance in the geographical distribution of research on recommender systems and digital nudges aimed at tackling overweight and obesity prevention [13]. High-income countries dominate the field, with the United States, Germany, the Netherlands, Belgium, Italy, and Portugal among the most represented. This distribution indicates that most research is focused in already technologically advanced regions, leaving middle- and low-income countries underrepresented. This discrepancy reflects broader socioeconomic disparities, including access to funding, infrastructure, and technology necessary to conduct such research. Expanding research efforts to include middle- and low-income countries would help to understand how digital interventions function across diverse socioeconomic contexts. Such inclusivity is crucial, as health behaviors such as diet and PA are global concerns, and solutions tailored to high-income settings may not be as effective in other contexts. In addition, overweight and obesity rates are high in many low- and middle-income countries [17]. A focus on a few countries hinders the adaptation of the identified digital nudges and recommender systems to other cultural, normative, and geographical contexts, thereby limiting the generalizability of the findings [1].

Regarding the second objective—classifying these systems based on the target population, behavior, personalization, and delivery—it was found that all recommender systems reported in the scoping review offered personalized recommendations. In this context, the findings of the scoping review differ from those reported in the literature. Bondevik et al [1], in their review of food recommender systems, state that 50.7% of the included recommender systems are not personalized. This discrepancy may be attributed to the slightly different scope of the scoping review. The present review only includes studies that explicitly evaluated the systems on users, which may result in a higher rate of personalization.

While many articles on recommender systems focus primarily on technical features [1,2,28,36], a central challenge identified in this scoping review is the lack of a clear and consistent definition of recommender systems, particularly in their application to health behaviors. Defining what constitutes an “actual system” can be ambiguous. Many studies describe systems that support users in selecting from a large pool of options without explicitly naming the system’s mechanisms for item retrieval. This difficulty in identifying recommender systems stems from insufficient descriptions in the literature, limiting the reproducibility and understanding of how these systems function. Future research should aim for more transparent reporting, providing clear system descriptions and code access [1]. This would also enable research to transition the tested systems into user studies—a gap also identified in this work. Currently, systems are developed and evaluated for accuracy, user-friendliness, and applicability [1,2]. However, real-world intervention and implementation evaluation with repeated measurement over a longer time and larger sample sizes are needed [2]. Also, studies testing the same recommender systems in different target groups are missing. Both would increase the generalizability of the results. This is consistent with the result that 54 articles did not mention the intended target group. The evaluations, however, were reported very heterogeneously, often with small sample sizes of adults (18-65) or younger persons (18-40). Further studies are needed to combine explicitly and test recommender systems and nudging interventions in robust user studies (including trials, mixed methods approaches, and interdisciplinary research) to identify approaches with the potential for a long-term positive impact also requested by El Majjodi et al [119].

Despite the potential for digital nudges to enhance adherence to recommender systems, this scoping review reveals that many systems fail to capitalize fully on nudges, which aligns with Jesse et al [7], who reported that 69 of 87 nudging mechanisms identified in the literature have not yet been tested. Visualizations of behaviors, such as calorie consumption or activity levels, are commonly used; however, they are often disconnected from established nudging strategies. More explicitly, integrating nudges into these systems allows users to be more effectively influenced toward healthier behaviors. For instance, intuitive graphics, personalized comparisons, and progress summaries can serve as subtle nudges that reinforce positive behavior change. However, these approaches often lack a connection to digital nudging research [7,120]. By leveraging these approaches more purposefully within design feedback tools, these systems could achieve greater user engagement and more impactful behavior change [121]. Future research should focus on developing methods that incorporate these insights to maximize the impact of recommender systems in health interventions. Furthermore, beyond health behaviors, recommender systems have a broad potential to contribute to sustainability and well-being goals, as noted in recent studies [122,123]. For example, recommender systems could help optimize energy consumption in light of sustainability and environmentally friendly lifestyle choices [124,125]. More research is needed to harness this potential to fully integrate behavioral insights into recommender systems and increase the selection share of sustainable and healthy items and to support behavior [126-129]. This must be accompanied by qualitative research addressing accessibility questions, balance (eg, healthy choices vs occasional indulgences), when the systems become intrusive, and how to balance taste preferences with health and environmental considerations—this may require perhaps options to preselect the main foci. El Majjodi et al [110] highlighted a crucial point that has often been overlooked in many system developments: the inherent capacity of these systems to learn. This could result in learning effects that deviate in undesirable directions. For example, systems might learn users’ tastes and recipe preferences, which, over time, may no longer align with healthy options. As the systems evolve based on the prioritization of past preferences, their parameters shift accordingly. Currently, there is no research or discussion on how such developments are monitored or compared against the initial preferences entered by users and what the user accepts.

Traditional evaluations of recommender systems have primarily focused on prediction quality and user satisfaction, utilizing a combination of online and offline evaluations, as also found in the included studies [1]. However, as Atas et al [130,131] point out, there are other critical dimensions to consider, such as how users perceive the recommended items or the visibility of certain items in recommendation lists. Atas et al [130] argue that preferences are unstable and subject to change during the recommendation process. These factors directly influence user behavior and the effectiveness of the systems in guiding healthier choices. Future evaluations should adopt a more holistic approach that includes these additional metrics, ensuring that recommender systems are accurate and effective in shaping user behavior toward positive outcomes.

### Implications for Research

This paper presents the current state of research on digital nudges and recommender systems for obesity prevention, highlighting important gaps in evidence and practice. While both approaches might be effective, the current evidence is mixed. Provided that methodology and evaluation standards are harmonized, and stronger interdisciplinary collaboration between intervention experts, user experience scientists, ethicists, and implementation researchers is established, these technologies can be applied in a more targeted and sustainable manner. This would also enable comparative effectiveness trials to provide stronger evidence on the effectiveness of these approaches in this field. Knowing about these gaps is crucial because it enables researchers to target their efforts where they can make the greatest impact and helps avoid repeating ineffective approaches or overlooking promising new directions. This scoping review identified various points that should be targeted in the future:

First, there is a pressing need to harmonize the definitions of recommender systems, interventions, digital nudges, and evaluation methodologies used in obesity prevention research. Clearer definitions and standards for system properties, targets, and evaluation foci will make studies more comparable and cumulative [126,132].

Second, advancing this field will require genuine interdisciplinarity, integrating research and expertise from intervention development, user experience (UX) science, ethics and data governance, implementation, and behavioral science. The collaboration can enhance the design and effectiveness of digital nudges and recommender systems and ensure that interventions are contextually appropriate, user-centered, and scalable in real-world settings. Combining intervention development and UX research addresses usability, engagement, satisfaction, and adaptation of systems for diverse populations based on rigorous behavioral theory and empirical testing. Implementation science frameworks help to evaluate how well these interventions are adopted, reach their intended audiences, and sustain their impact beyond pilot trials. Insights from behavioral science, such as optimal design, timing, and frequency of triggers, reminders, labels, and nudges, are indispensable for the technical aspects. By systematically connecting these disciplines, future research can address not only technological and efficacy questions but also barriers to adoption, ethical data use, and the real-world conditions necessary for population-level obesity prevention [132].

Third, current systems frequently use static user preferences but rarely leverage the learning capability and dynamic adjustment demonstrated in commercial products. Real-time personalization, adaptation, and interconnection among users remain underutilized in research interventions, though they are increasingly utilized commercially. A critical discussion must also address how commercial products that already integrate these advanced features could be leveraged for research purposes without compromising personal data ownership, trust, transparency, and adherence to ethical standards. Balancing the use of commercially developed technologies with robust protections for user privacy and data governance is crucial to maintaining public trust and meeting high ethical standards in health research and intervention.

Fourth, data governance, privacy, and user ownership of data were largely absent from the reviewed studies. Yet, these are central to building trust in future health technologies and ensuring ethical and legal compliance in the dissemination of expanded systems. This includes establishing clear standards and best practices for the transparent use of data, informed consent, secure data storage, and providing users with meaningful control over their personal information. Collaborations among ethicists, legal experts, technology developers, and end-users are essential to co-create solutions that foster trust, comply with evolving regulations, and enable responsible innovation and research. It is essential to highlight the positive aspects of the systems but also to address areas of risk, such as developers’ underlying assumptions, backward learning, and echo chambers. Such discussions are rarely included in current work.

Fifth, the scoping review predominantly focused on individual-level interventions. However, individual prevention alone cannot realize its full potential without supportive environments and population-level policy frameworks that enable healthy choices and prevent weight gain at scale. Population-level approaches are essential because they create the social, economic, and physical contexts necessary for sustainable behavior change. Integrating individual-level digital interventions, such as nudges and recommender systems, within broader population health policy approaches might be a long-term goal. This integration requires developing and evaluating multilevel interventions that consider policy, environmental, community, and health care system factors alongside personalized behavior change tools. By bridging individual and population approaches, research can better address the complex determinants of obesity and enable impactful, equitable prevention strategies at scale. This approach is a very complex one and needs, as a starting point, evidence-based and effective interventions.

### Limitations

Several limitations emerged during this review. First, many studies were excluded because they did not test their recommender systems in real-world settings, focusing instead on hypothetical scenarios or predefined datasets. This exclusion limits the generalizability of the findings, as it is unclear how these systems would perform in practical applications. Additionally, we excluded studies that did not label their nudging approach as digital nudges. This decision likely led to the exclusion of some studies that might have been relevant to our analysis. For instance, studies focusing on shopping behaviors or the use of labels in online stores may have used digital nudging techniques or interventions in the digital environment but were not explicitly categorized as such. Consequently, these studies might have been overlooked in our scoping review. This limitation underscores the importance of how nudging interventions are described and classified in research, as it impacts the comprehensiveness of evaluations in the field. A potential limitation of our scoping review is the identification of SB within the broader context of PA. In our scoping review, we directly searched for SB to identify and include studies addressing SB. We only found one study that specifically addressed SB. While SB can be considered part of a continuum of movement behavior, some studies may have indirectly included it without explicitly mentioning or assessing it as a distinct factor. Many studies likely focused solely on moderate to vigorous PA and consequently overlooked SB, one of the continuum’s extremities. This oversight is significant in light of ongoing debates regarding whether SB should be viewed as part of the movement behavior spectrum or as a separate entity [41,133]. Many publications fail to address SB independently, which can lead to a lack of emphasis on understanding its uniqueness and implications. Therefore, future research should explicitly consider and differentiate SB from other forms of PA to ensure a comprehensive understanding of movement behaviors and their impacts.

Another limitation is that we did not conduct a formal quality assessment. Scoping reviews are designed to map the extent and nature of available research rather than critically appraise the quality of the evidence, as would be the focus of a systematic review. Given the significant heterogeneity in methodologies, outcomes, and study designs, applying a uniform quality appraisal would not have been feasible without the risk of oversimplifying the diverse research landscape. Consequently, while this scoping review highlights the breadth of existing literature and its current gaps, the lack of quality assessment may affect the generalizability of the findings.

In line with the nature of scoping reviews as outlined above, this scoping review aims to provide a comprehensive overview of the existing literature in a particular research area, without focusing on specific outcome effects. The diverse range of topics and breadth of evidence precluded a detailed methodological quality assessment, which, as stated, might not have been feasible or appropriate given the exploratory aims of this scoping review. The broad scope and diversity of included studies make it challenging to report effect sizes or significances, leading to a qualitative categorization of outcomes as positive, negative, or mixed. This categorization choice is aligned with the scoping review’s objective to include studies presenting both qualitative and quantitative findings, further emphasizing potential limitations in generalizability and effect estimation. The primary focus remains on identifying key concepts and knowledge gaps rather than evaluating the effectiveness of specific interventions, thus necessitating caution when applying these findings to specific contexts or populations.

Additionally, the review revealed that many studies do not report essential system details, such as how they personalize recommendations or use user data, making it challenging to assess the full scope of their effectiveness. Future research should prioritize real-world testing and comprehensively describe system design, personalization, and interconnection mechanisms to enhance transparency and reproducibility.

A final consideration concerns the timing of our database search, which was completed in October 2023 and may, therefore, not fully reflect the most recent developments in the field, particularly regarding AI and machine learning. While generative AI and large language models have attracted increasing attention during this period, our search strategy focused on applications evaluated in human populations rather than on studies using predefined datasets. Consequently, some of the most recent innovations may not yet be represented among the studies identified. Future reviews could incorporate emerging research on generative AI and large language models to capture these dynamic developments more fully.

### Conclusion

Combining digital nudges and recommender systems can play a crucial role in overweight and obesity prevention by encouraging healthier lifestyle choices and subtly, nonintrusively guiding users toward better nutrition, PA, and SB habits. However, their effectiveness and, in particular, which mechanisms may be helpful have not yet been fully explored. More details on definitions and mechanisms, as well as more inclusive geographical representation, intervention and implementation tests, and user evaluation studies, are essential for advancing the field. Previous research has often focused on individual-level interventions; however, for a tangible impact at the population level, integration into multilevel approaches, including policy and environmental strategies, is essential. The personalized, dynamic adjustment of nudges in combination with recommender systems, alongside assurances of data privacy and user trust, offers promising avenues for future investigation. This underlines that the contribution of this review is not merely descriptive but emphasizes the advancement of interdisciplinary methodology, evaluation frameworks, and scalability of digital interventions as critical priorities for ongoing research. By addressing these challenges, future studies can better harness the potential of these systems to improve health outcomes and contribute to broader goals such as sustainability and well-being.

## Supplementary material

10.2196/73151 Multimedia Appendix 1 Search query.

10.2196/73151 Multimedia Appendix 2 Table S1: Study characteristics.

10.2196/73151 Checklist 1 PRISMA-ScR checklist.

## Abbreviations

PA: physical activity
PCC: Population, Concept, Context
PRISMA-ScR: Preferred Reporting Items for Systematic Reviews and Meta-Analyses extension for Scoping Reviews
SB: sedentary behavior
