# Supplementary material for: Mapping Digital Nudges and Recommender Systems for Obesity Prevention: Scoping Review
Source: Interact J Med Res. 2026 Apr 17;15:e73151. doi: 10.2196/73151 (PMC13089671; doi:10.2196/73151)
Supplement: Multimedia Appendix 1 [file ijmr-v15-e73151-s001.docx]

## Appendix 1

**Search Query**

MEDLINE(R) ALL 1946 to October 12, 2023 via Ovid
Searched/exported 13.10.2023

| **Search line** | **Search terms** | **Results** |
| --- | --- | --- |
|  |  |  |
| 1 | "recommend* system?".mp. | 1.108 |
| 2 | (digital adj2 nudg*).mp. | 23 |
| 3 | overweight.ti,ab. | 88.457 |
| 4 | obes*.ti,ab. | 379.222 |
| 5 | adipos*.ti,ab. | 133.189 |
| 6 | (weight adj3 (bod* or health* or unhealth* or gain* or chang* or retention or loss* or management)).ti,ab. | 418.074 |
| 7 | (physical adj3 (activit* or exertion? or training or inactivit*)).ti,ab. | 171.194 |
| 8 | (sedentary adj2 (behavior* or behaviour*)).ti,ab. | 9.873 |
| 9 | exp obesity/ | 263.306 |
| 10 | exp overweight/ | 274.760 |
| 11 | exp exercise/ | 249.036 |
| 12 | exp "sedentary behavior"/ | 13.717 |
| 13 | or/1-2 | 1.131 |
| 14 | or/3-12 | 1.137.336 |
| 15 | (food* or nutrition* or diet*).ti,ab. | 1.381.412 |
| 16 | exp food/ | 1.483.142 |
| 17 | exp diet/ | 331.137 |
| 18 | or/15-17 | 2.547.972 |
| 19 | 13 and (14 or 18) | 89 |
