# Supplementary material for: Mapping Digital Nudges and Recommender Systems for Obesity Prevention: Scoping Review
Source: Interact J Med Res. 2026 Apr 17;15:e73151. doi: 10.2196/73151 (PMC13089671; doi:10.2196/73151)
Supplement: Multimedia Appendix 2 [file ijmr-v15-e73151-s002.docx]

## Appendix 2

Table S1: Study characteristics

|  | **Characteristics** |  | |  | |  | |  | |  | **Evaluation** | |  |  | |  |
| --- | --- | --- | --- | --- | --- | --- | --- | --- | --- | --- | --- | --- | --- | --- | --- | --- |
|  | **Author, Year, Country, Population (specified in the Abstract) Behaviour targeted** | **Recommender System specified**  **Delivery channel** | | **System features reported** | | **Data collected** | | **Nudges** | | **Data used for personalisation**  **Interconnection** | **Evaluation aim** | | **Sample size**  **Gender**  **Age of Evaluation group** | **Any information about evaluation design/ evaluation tools** | | **Evaluation time if reported**  **Outcome** |
| **Recommender System**  **Nutrition** | | |  |  |  | |  | |  | |  |  | | |  | |
| 1 | Abbas et al., 2021 USA not specified nutrition (macronutrient composition) | case-based reasoning recommendation approach with diversity-focused dynamic critiquing  web | | recipe list with expanded view (information about the recipe) | | demographic information (age, gender, and education), online behaviour while looking for recipes | | n/a | | own  n/r | effectiveness evaluation | | 21-30  mixed  18-40 | pre-post survey | | positive |
| 2 | Abdool et al., 2015 Trinidad and Tobago not specified nutrition (meal choice) | case-based reasoning (CBR)  app | | food preferences | | food preferences | | n/a | | own  n/r | user satisfaction | | 21-30  mixed  18-40 | user survey with ratings on a scale of 0-10 | | positive |
| 3a | Ahn et al., 2015 USA not specified nutrition (shopping) | not specified  app + augmented reality (AR) | | image-based positioning, localising the user within the Grocery Aisle, AR-based user Interface, health-based grocery recommendation | | ambient (GPS, user health profile (allergies), information about grocery items | | n/a | | own  n/r | system validation | | 11-20  n/r  adults (18-65) | in-person survey design, questionnaire, interview | | positive |
| 3b |  |  | |  | |  | |  | |  | user satisfaction | | 101-200  n/r  adults (18-65) | questionnaire | | positive |
| 4 | Calvaresi et al., 2022 Switzerland not specified nutrition (macronutrient composition) | agent-based chatbot platform  n/r | | meal tracking (i.e., food image and barcode recognition), weight tracking, statistical evaluation and visualisation of the collected data, direct feedback | | n/r | | n/a | | own  no | system performance and utilisation | | 11-20  mixed  adults (18-65), older adults (65+) | questionnaire and generated logs | | 12 days  mixed |
| 5a | Castiglia et al, 2022 Italy not specified nutrition (macronutrient composition) | conversational food recommender system  n/r | | chatbot (pur textual, multi-modal (text plus visuals, multi-modal supplemented with nutritional labelling) | | food category, dietary constraints, preferences | | n/a | | own  no | choice satisfaction | | 101-200  n/r  n/r | recipe scoring, questionnaires | | positive |
| 5b |  |  | |  | |  | |  | |  | system evaluation | | 101-200  n/r  n/r | recipe scoring, questionnaires | | positive |
| 6 | Chao et al., 2020 USA not specified nutrition (meal choice) | not specified  app | | choice-based user interface (layout, nutrition information) | | n/r | | n/a | | own  n/r | user perception | | 11-20  n/r  18-40, 60+ | 2x2 full factorial experiment, questionnaire, person interview, think aloud | | no difference |
| 7a | De Pessemier et al., 2023 Netherlands not specified nutrition (meal choice) | Implicit ALS algorithm  app | | individual and group recommendations | | user preferences, allergies, diets, ratings, allergies disliked ingredients | | n/a | | own  n/r | user preference | | 11-20  mixed  18-40 | focus group | | positive |
| 7b |  |  | |  | |  | |  | |  | user experience | | 51-100  n/r  n/r | within-subjects experiment, App use, questionnaire (rating) | | positive |
| 8a | Elahi et al., 2015  Italy  not specified  nutrition (meals choice) | not specified  app | | interaction design, ratings and tacks of recipes | | short- and long-term preferences, ingredients | | n/a | | own  n/r | user satisfaction | | 11-20  mixed  adults (18-65) | questionnaire | | positive |
| 8b |  |  | |  | |  | |  | |  | system usability | | 11-20  mixed  adults (18-65) | questionnaire | | positive |
| 9a | Elsweiler et al., 2017 Germany not specified nutrition (meal choice) | not specified  n/r | | replacement recipes | | food preferences, enjoyment of cooking, frequency of online recipe site use | | n/a | | own  n/r | user satisfaction | | 101-200  mixed  18-40 | rating | | negative |
| 9b |  |  | |  | |  | |  | |  | performance evaluation | | 101-200  mixed  18-40 | rating | | positive |
| 9c |  |  | |  | |  | |  | |  | prediction evaluation | | 101-200  mixed  adults (18-65) | rating | | positive |
| 10 | Garcia et al., 2019 Philippines not specified nutrition (macronutrient composition) | not specified  app +web | | meal planner applications for tailored diet plans | | n/r | | n/a | | own  n/r | performance evaluation | | 21-30  n/r  n/R | task performance, rating | | positive |
| 11a | Garcia et al., 2021 Philippines not specified  nutrition (macronutrient composition) | not specified + rule-based reasoning  n/r | | recommendation of tailored meal plans | | knowledge, recommendation, and nutrition care, personalisation variables (e.g., preferences, restrictions, and personal goals) | | n/a | | own  n/r | system testing (Acceptability, usability, quality) | | 301-  n/r  18-40 | convergent parallel mixed-methods design, questionnaire | | positive |
| 11b |  |  | |  | |  | |  | |  | usability | | 301-  n/r  18-40 |  | | positive |
| 11c |  |  | |  | |  | |  | |  | quality | | 301-  n/r  18-40 |  | | positive |
| 12 | Grace et al., 2022 Australia not specified nutrition (meal choice) | content-based filtering  app | | recipe list | | meal and ingredient preferences, meals for cooking, review cooked meals | | n/a | | own  n/r | performance evaluation | | 41-50  mixed  adults (18-65) | semi-structured interviews | |  |
| 13 | Gutiérrez et al., 2017 Belgium not specified nutrition (shopping) | content-based + similarity index  desktop+web application | | head-mounted display for recognising print labels, barcodes, visualisation of similar products, impact on health, nutrients | | n/r | | n/a | | n/r  n/r | perceived quality | | 11-20  mixed  18-40 | offline evaluation | | mixed |
| 14a | Hafez et al., 2021 Egypt not specified nutrition (shopping) | hybrid  desktop | | clear images, allergens’ properties (e.g.: product includes (flour, eggs, water, nuts, and salt)), characteristics of the nutritional table (e.g., carbohydrates, dietary fibre, a percentage of saturated fat, good fat, protein, salt) | | n/r | | n/a | | n/r  n/r | effectiveness evaluation | | 51-100  n/r  n/r | online survey | | positive |
| 14b |  |  | |  | |  | |  | |  | performance evaluation | | 51-100  mixed  n/r | online survey | | positive |
| 15 | Hamdollahi Oskouei et al., 2022  Iran  not specified  nutrition (meal choice) | deep learning-based food recommender system  n/r | | comprehensive set of numerical, textual, and visual features | | comprehensive set of characteristics and features of users and foods, including users’ long-term and short-term preferences, users’ health conditions, demographic information, culture, religion, food ingredients, type of cooking, food category, food tags, diet, allergies, text description, and images of the foods | | n/a | | own  n/r | performance evaluation | | 11-20  mixed  n/r | not specified | | positive |
| 16 | Hauptmann et al., 2020 Netherlands not specified nutrition (macronutrient composition) | content-based filtering  app | | automated personalised visual feedback (nutrients, energy, statistics. simulation of future intake) and recommendations (recipe list (recipe list, explanation, ingredients, instruction) split by meal type) | | individual dietary behaviour, phenotype, and preferences | | n/a | | own  n/r | user impact evaluation | | 31-40  n/r  adults (18-65) | effect differences between group and effects within subjects, questionnaires, system tracking, interviews | | 2-3 months  positive |
| 17 | Khan et al., 2019 Ireland not specified  nutrition (meal choice) | hybrid  web | |  | | taste, demographics, costs, preferences | | n/a | | own  n/r | system evaluation | | 41-50  mixed  adults (18-65) | rating | | positive |
| 18 | Khan et al., 2021 Ireland  not specified  nutrition (meal choice) | hybrid  web | |  | | user preference | | n/a | | own  n/r | performance evaluation | | 41-50  mixed  adults (18-65) | rating | | Positive |
| 19 | Leipold et al, 2018 Germany not specified nutrition (macronutrient composition) | knowledge-based, personalised nutrition recommender system  app | | personalised persuasive features (visual feedback: Nutrient details screen, nutrient overview and statistics overview, with colour coding along traffic light warning, also used for recipes) | | users intake history, gender, age, BMI | | n/a | | own  n/r | system test | | 11-20  mixed  adults (18-65) | questionnaire, anthropometry | | 3 weeks  mixed |
| 20 | Massimo et al., 2017 Italy not specified nutrition (meal choice) | not specified  app | | recipe recommendation, tacking options | | age, gender, preferences, recipe ratings+explanation of motivation for the ratings | | n/a | | own  n/r | user satisfaction | | 41-50  n/r  n/r | rating | | positive |
| 21 | Musto et al., 2020  Italy  not specified  nutrition (meal choice) | knowledge-based recommender system  web | | n/r | | mood, knowledge (cooking experience), behavioural data (level of PA), health data (food requirements, amount of sleep, mood, stress level, weight (BMI)) | | n/a | | own  n/r | user satisfaction | | 101-200  n/r  n/r | web-based experiment  rating | | mixed |
| 22 | Musto et al., 2021 Italy not specified nutrition (meal choice) | hybrid  n/r | | n/r | | demographics (gender, age, height, weight), preferences (food, restrictions (lactose-free, vegan, etc.), goals), affect (mood), behavioural (level of PA), health data (lifestyle, BMI, amount of sleep, stress),  knowledge (cooking experience, available time, cost constraints) | | n/a | | own  n/r | effect evaluation | | 301-  mixed  adults (18-65) | online user study, rating | | positive |
| 23 | Norouzi et al., 2018 Iran Diabetic patients nutrition (meal choice) | knowledge-based recommender system  app | | snacks based on patient conditions and preferences, reminder | | PA, calories, patient profile (age, sex eight, birth date, arm and waist circumference, medication regimen and BMI as estimated by the system), recoding users favourites, lab results | | n/a | | own  n/r | performance evaluation | | 21-30  mixed  adults (18-65), older adults (65+) | ratings | | positive |
| 24 | Ozeki et al., 2022 Japan not specified nutrition (meal choice) | not specified  web | | recipes, favourite ingredients,  recommendations based on target nutrient intakes, visualisation of nutrition balances with selected recipes | | eating habits, user preferences, nutrient balance, number of recipes per day | | n/a | | own  n/r | performance evaluation | | 11-20  mixed  18-40 | systems log | | positive |
| 25a | Pecune et al., 2021  UK  not specified  nutrition (meal choice) | not specified + conversational agent  webpage with chat or button mode | | recipes recommendation, option to interact with the system | | user (specific foods), user constraints (healthiness, how healthy you would like to be, how hungry are you, diet/intolerance, time to cook, ingredients preference) | | n/a | | own  n/r | user perception (conversational skills) | | 101-200  mixed  adults (18-65), older adults (65+) | 2 × 2-between subject design, questionnaire | | positive |
| 25b |  |  | |  | |  | |  | |  | perceived quality of the recommendations | | 101-200  mixed  adults (18-65), older adults (65+) | system log, questionnaire | | positive |
| 26a | Ribeiro et al., 2017 Portugal older adults nutrition (macronutrient composition) | content-based filtering  mobile | | calculation of nutritional requirements, selection of food items for each meal and scaling the meals to match the user's caloric needs, meal plan, food tracking, grocery list, activity monitoring | | personal information (e.g. anthropometry, preferences), activity data through Fitbit devices | | n/a | | own  n/r | suitability | | 11-20  mixed  older adults (65+) | questionnaire | | mixed |
| 26b |  |  | |  | |  | |  | |  | Usability | | 0-10  n/r  older adults (65+) | task completion | | mixed |
| 27 | Samagaio et al., 2021 Portugal older adults nutrition (meal choice) | not specified + chatbot  n/r | | chatbot | | n/r | | n/a | | own  n/r | usability | | 21-30  n/r  n/r | questionnaire | | positive |
| 28a | Schäfer et al., 2019 Germany not specified nutrition (macronutrient composition) | not specified + rasch-based tailoring  app | | dietary tracking, visual feedback (calorie overview, nutrient details, nutrient overview, nutrient statistics, preferences profile, recommendation), personalised recipe recommendation | | eating habits activity habits, data on physiological measures (e.g. height, weight, age, gender, etc.) | | n/a | | own  n/r | performance evaluation | | 101-200  mixed  adults (18-65), older adults (65+) | mixed within and between subject design system logs, questionnaire | | 2 weeks  positive |
| 28b |  |  | |  | |  | | n/a | | own  n/r | behaviour change | | 101-200  n/r  adults (18-65), older adults (65+) | mixed within and between subject design, system log, questionnaire | | 2 weeks  positive |
| 28c |  |  | |  | |  | | n/a | | own  n/r | user perception | | 101-200  n/r  adults (18-65), older adults (65+) | mixed within and between subject design, system log, questionnaire | | 2 weeks  positive |
| 29 | Showafah et al., 2021  Indonesia  Babies and mothers  nutrition (macronutrient composition) | knowledge-based + TOPIS + Naive Bayes  n/r | |  | | parents | | n/a | | own  n/r | user satisfaction | | 0-10  woman  n/r | questionnaire | | mixed |
| 30 | Sookrah et al., 2019 Mauritius hypertensive patients nutrition (macronutrient composition) | content-based filtering  app | | user profile, menu plan, Monitoring BP levels | | user profile, age, food preferences, allergies, alcohol, smoking, BP level, dietary intake | | n/a | | own  n/r | user acceptance test | | 0-10  n/r  40-65 | system performance, questionnaire | | 1 week  mixed |
| 31 | Starke et al, 2021 (Serving) Netherlands not specified nutrition (meal choice) | collaborative filtering  app | | displayed five different recommendation lists with feature-based explanations (e.g., ‘Similar, but with fewer calories’), with the following re-sorting criteria | | n/r | | n/a | | own  n/r | user evaluation | | 301-  n/r  18-40 | 2 (single list vs multiple lists) x 2 (without or with explanations) between-subject user study, rating, scores | | positive |
| 32a | Starke et al., 2023 Netherlands not specified nutrition (meal choice) | content-based filtering  app | | demographics, self-reported healthiness, cooking experience | | n/r | | n/a | | own  n/r | user evaluation | | 301-  mixed  adults (18-65) | 2 (single-vs multi-list) x 2 (with or without explanations) between-subject design | | negative |
| 32b |  | knowledge-based filtering  n/r | | personal information and preferences (e.g., weight status, cooking experience) | | n/r | | n/a | | own  n/r | user evaluation | | 101-200  mixed  18-40 | 2 × 2-between user design | | negative |
| 33 | Svensson et al., 2003 Sweden not specified nutrition (meal choice) | collaborative filtering  n/r | | n/r | | n/r | | n/a | | n/r  n/r | system evaluation | | 51-100  n/r  n/r | Questionnaire | | 6 months  positive |
| 34a | Thongyoo et al., 2020 Thailand diabetic persons nutrition (meal choice) | not specified+chatbot  mobile | | food recommendations, nutrition information, advice, Reports | | demographic data including age, weight, height, and gender to calculate, their BMI, reference a specific dietary criterion | | n/a | | own  n/r | user satisfaction | | 21-30  mixed  15-17, adults (18-65), older adults (65+) | online survey | | positive |
| 34b |  |  | |  | |  | |  | |  | chat performance evaluation | | 21-30  Mixed  15-17, adults (18-65), older adults (65+) | system performance | | positive |
| 35a | Vandeputte et al., 2023 France not specified nutrition (meal choice) | not specified + artificial intelligence-based personalised suggestions  app | | n/r | | n/r | | n/a | | n/r  n/r | plausibility test | | 201-300  n/r  adults (18-65) | online experiment | | positive |
| 35b |  |  | |  | |  | |  | |  | choice satisfaction | | 21-30  n/r  adults (18-65) | system performance | | mixed |
| 36a | Yang et al., 2017  USA  not specified  nutrition (meal choice) | not specified + online learning algorithm + food image analysis tool  n/r | | simple quiz-based visual interface | | individuals’ nutritional expectations (health goals), dietary restrictions, and fine-grained food preferences, food restrictions | | n/a | | own  n/r | system evaluation | | 201-300  n/r  n/r | not specified | | positive |
| 36b |  |  | |  | |  | |  | |  | validation of feasibility and effectiveness | | 51-100  n/r  n/r | not specified | | positive |
| **Recommender System**  **Physical Activity** | | |  |  |  | |  | |  | |  |  | | |  | |
| 37 | Coppens et al., 2023 (Connectiong) Belgium not specified PA | not specified  app | | n/r | | sensor data, Ecological Momentary Assessments, mood before PA, situation, company, time, weather, step count history, location type history, momentary motivation for PA, mood after PA, motivation | | n/a | | own  n/r | effectiveness evaluation | | 31-40  n/r  adults (18-65) | pre-post questionnaires, EMA data | | 21 days  positive |
| 38 | Coppens et al., 2023 (Motivation) Belgium not specified PA | content-based recommender system  app | | recommended PA or tip, their own performed PA (“enter own activity”), or a reason why now is not a good time for PA (“not now”), notification with a tip  recommendation five times per day, | | user’s mood, preference history, and estimated current situation (e.g., work or free time), | | n/a | | own  n/r | effectiveness evaluation | | 21-30  n/r  n/r | RTC, ratings | | 8 weeks  positive |
| 39 | Coppens et al. 2023 (Analyzing) Belgium not specified PA | difference between content-based and user-based collaborative filtering algorithms | | select one of the three PA or three tip , or recommendations or add own PA | | n/r | | n/a | | own  n/r | system evaluation | | 21-30  n/r  n/r | between-subject study design, pre-test, post-test, questionnaires | | 8 weeks  mixed |
| 40 | He et al., 2014 USA not specified PA | not specified  app | | feedback, goal-setting, PA suggestions (taking into account weather conditions) | | continuous PA monitoring background of Smartphone, user goals, SB time is detected, time, location weather, personal information (schedule, fitness level) | | n/a | | own  n/r | evaluate the functionality and user experience | | 0-10  n/r  n/r | system performance, questionnaire | | 2 weeks  mixed |
| 41 | Hermanny et al., 2019  Germany  not specified  PA | not specified  app | | interface element design for planning of physical activity (interfaces: goal selection, conversion to time, active unit planning) goal striving (kilocalories (kcal) recommendation (number of steps, one of the most common units for measuring physical activity, time needed) threedifferent intensity levels (low, moderate, high)-> detailed planning | | user and context data | | n/a | | n/r  n/r | system and performance evaluation | | 21-30  n/r  adults (18-65), older adults (65+) | behavioural observation, think-aloud, online questionnaire | | positive |
| 42 | Lin et al., 2011 Netherlands not specified PA | not specified  web | | personalised and contextualised advice on PA -> messages on screen to which the user can respond | | GPS or GSM, user profile, personal agenda, weather, time | |  | | own  n/r | feasibility study | | 0-10  mixed  adults (18-65) | interviews, user data | | 5 weeks  mixed |
| 43 | Martinez et al., 2019 Portugal older adults PA | not specified  robot | | Robot, that can work without a therapist, based on exercise timetable, recommender, exercise descriptor, exercise recogniser, user feedback system | | user’s personal information (e.g., name, age, medical condition) and exercise information (e.g., difficulty, appropriateness, repetitions) and the caregiver’s information (e.g., exercise plans | |  | | own  n/r | system test | | 0-10  n/r  60+ | not specified | | positive |
| 44 | Ono et al, 2022 Japan not specified PA | not specified  app | | in combination with wearable | | priority, free time slot, total number of activities/day, ambient situation (weather), own situation (schedule) | |  | | own  n/r | performance evaluation | | 11-20  Mixed  18/40 | questionnaire, user data | | mixed |
| 45a | Sengupta et al., 2020 USA coronary heart disease patients PA | not specified + Internet of thinks  desktop+app+wearable | | goal setting, real-time progress monitoring, EMA surveys, videos | | patients’ daily physical activities, eating episodes, moods, environment, and so forth using sensors embedded inside the smartwatch provided to patients, and EMA surveys sent via a mobile application. | | n/a | | own  n/r | pre-programmed intervention messages | | 0-10  n/r  40-65, older adults (65+) | quasi-experimental design | | 13 weeks (91 days)  positive |
| 45b |  |  | |  | |  | |  | |  | role of EMA to increase PA | | 0-10  n/r  40-65, older adults (65+) | quasi-experimental design | | 13 weeks (91 days)  positive |
| 46 | Zhao et al., 2020  Canada  not specified  PA | hybrid with decision tree–based module  app+wearable | | personalised gamified activity recommendation, profile views (daily, weekly), Facebook view, hacker mode view, theme colour customisation view | | demographic (e.g., including age, gender, height, weight, number of hours they spend per week exercising), type of Android Wear owned, types and duration of playing video games (eg, PC, console, and mobile), in-game data with google analytics | | n/a | | own  n/r | effectiveness evaluation | | 31-40  Mixed  18-40 | not specified | | 60 days  positive |
| **Recommender System**  **Nutrition and Physical Activity** | | |  |  |  | |  | |  | |  |  | | |  | |
| 47a | Afonso et al., 2020 Portugal preschool children (3-6 years) nutrition (eating, drinking) PA, sleeping | automated filtering system  app | | questionnaire, educational videos | | health data (weight status, food/beverage intake, PA, sleeping, norms/attitudes of parents | | n/a | | own  n/r | adequacy of the recommendation system | | 31-40  Mixed  18-40 | questionnaire survey | | 3 months  positive |
| 47b |  |  | |  | |  | |  | |  | pilot study | | 11-20  Mixed  18-40 | RTC, questionnaire survey | | 3 months  positive |
| 48 | Alcaraz-Herrera et al., 2019 UK not specified (nutrition (meal choice), PA | evolutionary recommender system  n/r | | n/r | | (i) basic demographic information: gender and age; (ii) height and weight; (iii) food preferences (iv) exercise preferences (v) preferred intensity for exercises, vi) main fitness goal | | n/a | | own  n/r | online user-satisfaction evaluation | | 51-100  n/r  n/r | online survey | | positive |
| 49 | Hales et al., 2016  USA  adults with overweight/obesity  nutrition (macronutrient composition), PA | not specified  app | | notifications, theory-based messages | | calories tracking, weight, minutes of PA, age, race, gender, BMI, employment status, marital status, digital literacy | | n/a | | n/r  n/r | efficacy evaluation | | 51-100  Mixed  Adults (18-65) | RTC | | positive |
| 50 | Nam et al, 2015 South Korea not specified nutrition (macronutrient composition), PA | not specified  app | | a sensor infrastructure, context management, a recommendation system and a display (two modes - indoor and outdoor services; information on menus, prohibited foods, and the total caloric intake) | | height, weight, blood sugar, total cholesterol, blood pressure, and amount of daily caloric expenditure, blood pressure, blood sugar, and total cholesterol, | | n/a | | own  n/r | effectiveness evaluation | | 0-10  mixed  18-40 | not specified | | 20 days  positive |
| 51 | Palomares et al., 2022 UK not specified nutrition (meal choice), PA | evolutionary recommender system with integration of fuzzy inference engine  n/r | | individual recommendations (diet, PA) | | physical status information: gender, age, height, weight, activeness/passiveness level, (ii) physical activity habits, minutes per day and days per week, (iii) well-being goal (iv) explicit preferences over 14 categories of food, e.g. poultry, cereal, fish, etc.; (v) explicit preferences over eight categories of PA | | n/a | | own  n/r | system validation | | 101-200  n/r  n/r | not specified | | positive |
| 52a | Rabbi et al., 2015 USA not specified nutrition (meal choice), PA | not specified  app | | behaviour tracking (PA, diet), suggestion to change, possibility to remove suggestions | | preference | | n/a | |  | pilot evaluation | | 0-10  Mixed  n/r | food logs | | 3 weeks  positive |
| 52b |  |  | |  | |  | |  | |  | efficacy evaluation | | 11-20  mixed  adults (18-65) | single case experiment | | 14 weeks  positive |
| **Mixed**  **Recommender System and Nudges** | | |  |  |  | |  | |  | |  |  | | |  | |
| 53 | ElMajjodi et al., 2022 Norway not specified nutrition (meal choice) | singular value decomposition (SVD)  n/r | | recommender system, food labelling, personalisation | | age, gender, level of education, self-reported food-related behaviours (e.g., level of cooking experience, healthiness of eating habits, specific eating goals (e.g., eating less sugar),  dietary restrictions), preferred food categories | | labelling, personalisation | | own  n/r | effect evaluation | | 301-  mixed  18-40 | 2 (non-personalised vs. personalised recipe advice) x 3 (No Label, Multiple Traffic Light, Nutri-Score) between-subjects design, online survey, FSA score, questionnaires | | mixed |
| 54 | Starke et al., 2022 (Boosting) Netherlands not specified nutrition (meal choice) | comparison between systems  n/r | | n/a | | perception and experience aspects, user characteristics | | boost (labelling) | | own  n/r | effect of the boost | | 201-300  mixed  n/r | 2 × 2-between subject design, online evaluation | | Positive |
| **Recommender System and Game** | | |  |  |  | |  | |  | |  |  | | |  | |
| 55 | Cromjongh et la., 2023  Netherlands  not specified  PA | not specified + exergames  n/r | | n/r | | n/r | | n/a | | n/r  n/r | perception and attitude evaluation | | 301-  mixed  adults (18-65), older adults (65+) | online survey | | mixed |
| 56 | Giabbanelli et al., 2013 Canada adults nutrition (macronutrient composition) | not specified + health game  app+desktop | | n/r | | psychosocial and demographic characteristics | | n/a | | own  n/r | system test | | 11-20  woman  adults (18-65) | game play monitoring | | positive |
| 57a | Gomez-Del-Rio et al., 2020 Spain obese children healthy living (diet, PA) | hybrid+game  app+game | | n/r | | medical records, blood tests, anthropometric, biometric data from sensors, geolocalisation data, emotional data, behavioural data, diet data, PA data, social data, UX data, player profile, socioeconomic data, situational data, user preference, personal characteristics, environmental data | | n/a | | own  n/r | system validation | | 11-20  mixed  adults (18-65) | not specified | | 3 years  positive |
| 57b |  |  | |  | |  | |  | |  | UX Validation | | 0-10  mixed  adults (18-65) | rating | | positive |
| 57c |  |  | |  | |  | |  | |  | gamification validation | | 41-50  mixed  children (0-14) | questionnaire | | mixed |
| **Nudges** | | |  |  |  | |  | |  | |  |  | | |  | |
|  |  |  | |  | |  | |  | |  |  | |  |  | |  |
| 58 | Berger et al. 2020 Germany not specified nutrition (shopping) | n/a  online grocery store | | shopping list, product card, shopping card | | n/r | | default, simplification, social norm | | n/r  n/r | effectiveness evaluation | | 201-300  n/r  n/r | online field experiment, rating of food choice, questionnaire | | mixed |
| 59 | DeBauw et al., 2022  Belgium  not specified  nutrition (shopping) | n/a  mock-up online grocery store | | nudges/scores added to the grocery items | | gender, age, educational classes, household size, employment, retired, net household income per month | | product recommendation agents, product scores, a real-time average impact score of the chosen food basket and a personalised social norm | | n/r  n/r | effect evaluation | | 301-  mixed  adults (18-65) | RTC, focus groups | | mixed |
| 60a | DiCosola et al., 2022 USA not specified nutrition (shopping) | n/a  n/r | | n/r | | health, fitness, and nutrition | | social influence nudge (nutritional information nudges with added in-group (people like you) and out-group (people not like you) social comparisons) | | n/r  n/r | pilot experiment | | 51-100  n/r  n/r | online experiment | | positive |
| 60b |  |  | |  | |  | |  | |  | core experiment | | 301-  n/r  n/r | online experiment | | mixed |
| 61 | Haile et al., 2020  Scotland  working population  PA, SB, mental well-being | n/a  cross-platform personalised digital intervention | | n/r | | goal, environment, and preferences of nudges (i.e., what content the user likes/does not like to engage with), gender, age, height, weight, ethnicity, health status, working status, and occupation, user’s individual circumstances and preferences | | cards, messages, notifications (0% seated/standing exercises and stretches nudges, 14% relaxed breathing nudges, 11% screen breaks and/or improved work environment nudges, 8% mindfulness nudges, and 7% hydration nudges) | | n/r  n/r | pilot intervention test | | 41-50  mixed  adults (18-65) | trial, semi-structured interviews | | mixed |
| 62a | Jesse et al., 2021 Austria not specified nutrition (shopping) | n/a  web | |  | | food category, personal feedback, cooking, abilities, health orientation, age, gender | | highlighting, defaults, social information, warning, hybrid | | n/r  n/r | effectiveness of nudges | | 101-200  mixed  18-40 | exploratory study | | positive |
| 62b |  |  | |  | |  | |  | |  | validation of best-performing nudges | | 51-100  mixed  18-40 | rating | | positive |
| 63 | Michels et al., 2023 Germany not specified nutrition (meal choice) | n/a  mock-up online store | | shop items with different nudges | | n/a | | salience, transparency, self-nudging | | n/r  n/r | effect evaluation | | 301-  mixed  18-40 | RTC survey-based online experiment | | positive |
| 64 | Reinholdsson et al., 2021 Sweden not specified nutrition (meal choice) | n/a  digital menu card | | nudges/cues on the vegan menu | | n/a | | hedonic cue, menu position, warm-glow cue, and a descriptive norm | | n/r  n/r | performance evaluation | | n/a  n/a  n/a | large-scale field-experiment, sales data | | Monday-Friday, 5 weeks  mixed |
| 65a | Schomaker et al., 2022  Netherlands  not specified  nutrition (macronutrient composition) | n/a  n/r | | n/r | | unknown | | attentional bias induced by visual directional cues | | n/r  n/r | food choice experiment | | 201-300  unknow  adults (18-65) | online survey | | positive |
| 65b |  |  | |  | | age, gender, diet, weight, subjective personal health | |  | |  | stimuli evaluation | | 101-200  mixed  adults (18-65) | online survey, ratings | | positive |
| 66 | Starke et al., 2021 (nudged) Netherlands not specified nutrition (meal choice) | n/a  web application | | n/a | | n/r | | serial-position effect (ranking, personalisation) | | n/r  n/r | effect evaluation | | 11-20  n/r  18-40 | 2x2-within-subjects experiment | | mixed |
| 67 | Teuber et al., 2022 Germany students PA | n/a  MS Teams | | n/a | | n/r | | daily motivational prompts | | n/r  n/r | effectiveness evaluation | | 51-100  mixed  18-40 | RTC, questionnaire | | 10 days  no difference |
| 68 | Willinger et al., 2023 Germany patients with congenital heart disease PA | n/a  app | | n/a | | objective PA via wearable | | information nudge (daily SMS) | | n/r  n/r | intervention testing | | 51-100  mixed  15-17 | RTC, wearable | | 12 weeks  mixed |
